# Dose-dependent association of depression with incidence of end-stage kidney disease and all-cause mortality, CKD-JAC study: a retrospective cohort study

**DOI:** 10.1007/s10157-026-02908-8

**Published:** 2026-08-04

**Authors:** Maki Shinzawa, Kenta Murotani, Takahiro Imaizumi, Ryohei Yamamoto, Yoshitaka Isaka, Takashi Wada, Shoichi Maruyama

**Affiliations:** 1 Health and Counseling Center, The University of Osaka, 1-17 Machikaneyama-cyo, Toyonaka, Osaka 560-0043 Japan; 2 Department of Nephrology, The University of Osaka Graduate School of Medicine, Suita, Japan; 3 School of Medical Technology, Kurume University, Kurume, Japan; 4 Biostatistics Center, Kurume University, Kurume, Japan; 5 Department of Advanced Medicine, Nagoya University Hospital, Nagoya, Japan; 6 Department of Nephrology and Rheumatology, Kanazawa University, Kanazawa, Japan; 7 Department of Nephrology, Nagoya University Graduate School of Medicine, Nagoya, Japan

**Keywords:** Depression, End-stage kidney disease, All-cause mortality

## Abstract

**Background:**

Although depression is common among patients with chronic kidney disease (CKD), its impact on the incidence of end-stage kidney disease (ESKD) and all-cause mortality remains unclear.

**Methods:**

This retrospective cohort study used Cox proportional hazards (CPH) models and CPH models with penalized splines to assess the association between depression severity and the incidence of ESKD and mortality in patients with CKD, drawing on data from the Chronic Kidney Disease Japan Cohort (CKD-JAC) Study.

**Results:**

This study included 1,694 patients with CKD (median age 61 years; mean eGFR 29.0 ± 12.2 mL/min/1.73 m^2^; median Beck Depression Inventory-II (BDI-II) score 8). Depression severity was classified into four categories: normal (0–13), mild (14–19), moderate (20–28), and severe (≥ 29). In Multivariable CPH models, ESKD risk showed a stepwise elevation in hazard ratios (HRs) without reaching statistical significance, whereas, mortality risk increased significantly in the moderate and severe group: HR 3.11 (95%CI 1.61–6.03) and 3.51 (1.40–8.80). Additionally, CPH models demonstrated a dose-dependent association between BDI-II categories and the incidence of both ESKD and mortality. CPH Models with penalized splines further revealed a gradual increase in ESKD risk, and a J-shaped association in mortality risk.

**Conclusion:**

Depression severity showed dose-dependent associations with increased the incidence of ESKD and mortality in patients with CKD. Although mortality risk was significantly higher in the moderate and severe groups (BDI-II score ≥ 20), the stepwise elevation in ESKD risk did not reach statistical significance, and an optimal threshold for ESKD risk could not be identified.

**Supplementary Information:**

The online version contains supplementary material available at https://doi.org/10.1007/s10157-026-02908-8.

## Introduction

Chronic kidney disease (CKD), defined by an estimated glomerular filtration rate (eGFR) < 60 mL/min/1.73 m^2^ and proteinuria, affects approximately 10% of the global population, and the number of patients has risen in recent years [1, 2]. In 2017, an estimated 850 million individuals were living with CKD worldwide [3]. Major causes include diabetes, hypertension, primary and secondary glomerular disease, acute kidney injury, and aging [2, 3]. Patients with CKD are at high risk of progression to end-stage kidney disease (ESKD), cardiovascular disease (CVD), and premature mortality [4, 5]. As kidney function declines, the need for kidney replacement therapy (KRT) increases, imposing social and economic burdens and reinforcing CKD as a major public health and financial concern [6–8]. Established risk factors for ESKD progression include diabetes, hypertension, CVD, and unhealthy lifestyle habits such as smoking, short sleep duration, and poor sleep quality [9, 10]. The role of psychological risk factors in ESKD progression, however, remains poorly understood.

Depression, a prevalent mental health disorder and global public health concern, affects approximately 5% of the population [11, 12] as assessed by standardized tools such as the Beck Depression Inventory-II (BDI-II) [13] and the Patient Health Questionnaire-9 (PHQ-9) [14]. Depression is associated with adverse outcomes, including CVD, cancer, neurological disorders, and increased mortality [15]. Among patients with CKD, approximately 20% experience depression [16, 17], which has been linked to a 3.6-fold higher risk of all-cause mortality [18]. One potential mechanism is oxidative stress, which is linked to albuminuria in patients with CKD. Similarly, as observed in patients with CKD [19], the severity of depression is associated with albuminuria in the general population [20]. Notably, the relationship between depression and mortality risk may differ across racial and ethnic groups [21]. Additionally, several studies have explored the association between depression and CKD progression, particularly the initiation of KRT. Some reported a significant association [22–24], whereas other studies failed to confirm this association [25, 26], leaving the role of depression in ESKD development unresolved. Most prior studies analyzed depression as a binary variable, classifying patients as depressed (BDI-II ≥ 11) or not. This dichotomization may obscure gradations of depression severity and hinder the evaluation of dose-dependent associations with disease outcomes. In Japan, previous study has been limited to single-center investigations focusing primarily on diabetic kidney disease, with insufficient representation of nondiabetic CKD and inadequate statistical power [26]. Therefore, a large-scale, multicenter study is warranted to clarify the association between depression severity and CKD progression, including the initiation of KRT, in a Japanese population.

This retrospective cohort study aimed to assess the association between BDI-II score and the incidence of all-cause mortality and ESKD in 1,694 patients with CKD using Cox proportional hazards (CPH) models and CPH models incorporating penalized splines for BDI-II score. The findings clarify the clinical relevance of BDI-II scoring in predicting mortality and ESKD among patients with CKD.

## Methods

### Study design and participants

**Fig. 1:**
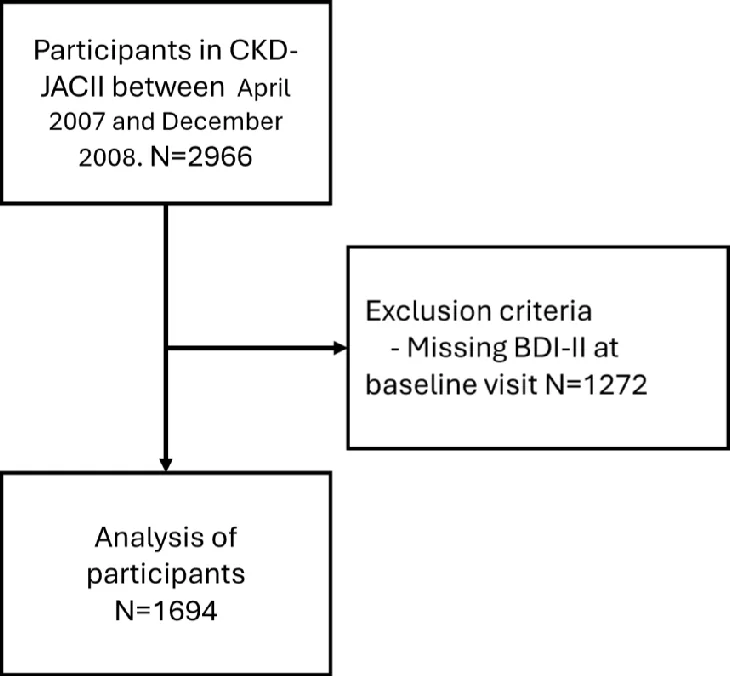
Flowchart illustrating the selection of study participants in the CKD-JAC Study

The Chronic Kidney Disease Japan Cohort (CKD-JAC) Study is a multicenter, prospective, observational cohort of 2,966 participants enrolled at 17 facilities across Japan between April 2007 and December 2008. Details of the protocol have been described elsewhere [27]. Briefly, eligible participants were aged 20–75 years with an eGFR of 10–59 mL/min/1.73 m^2^. eGFR was calculated using the following formula [28]: eGFR (mL/min/1.73 m^2^) = 194 × serum creatinine (mg/dL)^−1.094^ × age (years)^−0.287^ × 0.739 (if female). Exclusion criteria included polycystic kidney disease, HIV infection, liver cirrhosis, active cancer, or a history of transplantation or KRT. After obtaining informed consent, 2,966 participants were provided with self-administered, self-reported questionnaires to complete and return by mail. Completing the questionnaires was not mandatory for participants in the CKD-JAC study. After excluding 1,272 individuals (42.9%) due to missing BDI-II scores [29], the final cohort comprised 1,694 participants (57.1%) (Fig. 1).

This study was approved by the Ethics Committee of the Japanese Society of Nephrology (approval number: 45). It was conducted in accordance with the Japanese Ethical Guidelines for Medical and Biological Research Involving Human Subjects [30] and the Declaration of Helsinki [31]. Written informed consent was obtained from all patients.

### Measurement

The main exposure was depression severity, defined by BDI-II score. Participants were classified into four groups: normal (0–13), mild (14–19), moderate (20–28), and severe (≥ 29) [32]. Baseline variables included age, sex, body mass index (BMI), systolic and diastolic blood pressure (BP), eGFR, urinary albumin-to-creatinine ratio (UACR), educational background, annual income, smoking status, alcohol consumption, use of antidepressants, renin–angiotensin system inhibitors (RASi), and history of depression, diabetes mellitus (DM), hypertension, and CVD. Depression history was defined as a diagnosis of major depressive disorder or bipolar disorder within 12 months of baseline. CVD history included coronary and cerebrovascular disease, congestive heart failure, and peripheral arterial disease. RASi included angiotensin-converting enzyme inhibitors and angiotensin II receptor blockers. Educational background was grouped as low (less than secondary school), middle (completed secondary school), moderately high (some college), or high (completed college/graduate school). Annual income was categorized as low (< 3 million [M] Japanese yen [JPY]), middle (3–5 M JPY), moderately high (5–8 M JPY), or high (> 8 M JPY).

The primary outcome was time to all-cause mortality or ESKD, defined as initiation of KRT. Follow-up extended from baseline to the outcome, last visit, or administrative censoring (March 31, 2013, for one facility with 84 participants; June 30, 2018, for the rest of the clinical sites in the CKD-JAC Study).

### Statistical analysis

Baseline characteristics across BDI-II categories were assessed for trends using the Cochran–Armitage test for categorical variables and the Jonckheere–Terpstra test for continuous variables. For the Cochran–Armitage test, primary kidney disease was dichotomized into chronic glomerulonephritis versus other diseases; educational background and income into low/middle versus moderately high/high; and smoking and alcohol use into never vs current/past.

Cumulative probabilities of mortality and ESKD were estimated using Kaplan–Meier methods, and the dose–response relationship for depression severity was assessed using the log-rank trend test. The incidence of mortality and ESKD was analyzed using CPH models. In Model 1, facility-stratified CPH models were applied to account for heterogeneity in patient populations across facilities. Model 2 adjusted for age (years), sex, systolic BP (mmHg), BMI (Kg/m^2^), eGFR (mL/min/1.73m^2^), log UACR, and Model 1 covariates. Model 3 additionally adjusted for smoking status, antidepressant use, RASi, and history of depression, DM, and CVD; it included 1,674 participants due to missing smoking data. To evaluate the dose-dependent association between depression severity and outcomes in CPH models, we treated BDI-II categories as continuous variables. Proportional hazards assumption was examined by including interaction terms between the BDI-II score and the logarithm of survival time in the CPH model. None of the interaction terms were statistically significant, indicating no evidence of time-dependent effects. Therefore, the proportional hazard assumption was considered to be satisfied for the BDI-II score.

To examine potential non-linear relationships between BDI-II score and event risk, we fitted a multivariable CPH model with penalized splines (pspline function) for BDI-II score. This model had three knots at the 25th, 50th, and 75th percentiles of the BDI-II distribution, and it adjusted for the covariates in Model 3. This method flexibly estimates the functional form between BDI-II and the log hazard ratio (HR). Using the smoothHR package, we plotted the estimated log HR curve with BDI-II = 0 as the reference. The plot shows the log HR on the y-axis and BDI-II score on the x-axis, illustrating risk changes across the score range. To evaluate non-linearity, we compared a spline model with its corresponding linear model using a likelihood ratio test.

Continuous variables are reported as mean ± standard deviation (SD) or median with interquartile range (IQR), and categorical variables as counts and proportions. Statistical significance was set at P < 0.05 (two-sided). Analyses were performed using R version 4.2.1 (survival, smoothHR packages) and SAS version 9.4 (SAS Institute, Cary, North Carolina).

## Results

This study included 1,694 patients with CKD, with a median age of 61 years (IQR 52–69), an eGFR of 29.0 ± 12.2 mL/min/1.73 m^2^, and a BDI-II score of 8 (4–13). Table 1 summarizes baseline characteristics of the normal (n = 1,304), mild (n = 214), moderate (n = 132), and severe (n = 44) groups. The median BDI-II scores (IQR) were 6 (3–9), 16 (15–17), 23 (21–25), and 33 (30–36) for the normal, mild, moderate, and severe groups, respectively. Across BDI-II categories, stepwise associations were observed not only for antidepressant use and history of depression, but also for sex, eGFR, UACR, educational background, annual income, alcohol consumption, and history of DM and CVD.

**Table 1:** Baseline characteristics, stratified by BDI-II category

	BDI-II category				
	Normal (0–13) n = 1,304	Mild (14–19) n = 214	Moderate (20–28) n = 132	Severe (≥ 29) n = 44	P _trend_
BDI-II scores	6 (3–9)	16 (15–17)	23 (21–25)	33 (30–36)	< 0.001
Female, n (%)	376 (28.8)	90 (42.1)	63 (47.7)	17 (38.6)	< 0.001
Age, years	61 (52–69)	62 (52–68)	59 (50–67)	58 (53–68)	0.290
BMI, Kg/m^2^	23.6 ± 3.7	23.4 ± 3.9	22.9 ± 4.0	22.7 ± 4.7	0.051
Systolic blood pressure, mmHg	131 ± 18	133 ± 20	130 ± 19	126 ± 18	> 0.999
Diastolic blood pressure, mmHg	77 ± 12	77 ± 11	76 ± 13	72 ± 13	0.086
eGFR, mL/min/1.73m^2^	29.6 ± 12.0	28.9 ± 12.6	26.8 ± 13.2	28.2 ± 13.0	0.011
Urinary albumin creatinine ratio	475 (122–1191)	621 (239–1443)	628 (131–1474)	620 (54–1545)	0.008
Primary kidney disease					
Chronic glomerulonephritis	613 (47.0)	90 (42.1)	64 (48.5)	16 (36.4)	0.290
Diabetic nephropathy	199 (15.3)	57 (26.6)	29 (22.0)	12 (27.3)	
Nephrosclerosis	270 (20.7)	31 (14.5)	23 (17.4)	9 (20.5)	
Others	222 (17.0)	36 (16.8)	16 (12.1)	7 (15.9)	
Educational background ^a)^					
Low, n (%)	174 (13.5)	30 (14.2)	22 (17.1)	8 (18.6)	< 0.001
Middle, n (%)	484 (37.4)	98 (46.4)	58 (45.0)	19 (44.2)	
Moderately high, n (%)	216 (16.7)	41 (19.4)	23 (17.8)	8 (18.6)	
High, n (%)	419 (32.4)	42 (19.9)	26 (20.2)	8 (18.6)	
Annual income ^a*)*^					
Low, n (%)	184 (17.8)	54 (30.2)	35 (35.7)	18 (58.1)	< 0.001
Middle, n (%)	274 (26.5)	57 (31.8)	27 (27.6)	8 (25.8)	
Moderately high, n (%)	268 (25.9)	41 (22.9)	24 (24.5)	2 (6.5)	
High, n (%)	307 (29.7)	27 (15.1)	12 (12.2)	3 (9.7)	
Smoking status ^a)^					
Never, n (%)	671 (52.0)	112 (52.6)	71 (55.0)	19 (44.2)	0.919
Current, n (%)	229 (17.7)	38 (17.8)	24 (18.6)	14 (32.6)	
Past, n (%)	391 (30.3)	63 (29.6)	34 (26.4)	10 (23.3)	
Alcohol consumption ^a)^					
Non, n (%)	457 (36.1)	95 (45.7)	59 (46.1)	16 (36.4)	< 0.001
Current, n (%)	573 (45.3)	66 (31.7)	38 (29.7)	14 (31.8)	
Past, n (%)	235 (18.6)	47 (22.6)	31 (24.2)	14 (31.8)	
History of depression, n (%)	10 (0.8)	2 (0.9)	5 (3.8)	6 (13.6)	< 0.001
Use of antidepressants, n (%)	8 (0.6)	2 (0.9)	4 (3.0)	4 (9.1)	< 0.001
History of hypertension	1,216 (93.3)	203 (94.9)	127 (96.2)	41 (93.2)	0.250
Use of Ace inhibitors and ARB, n (%)	1079 (82.7)	181 (84.6)	112 (84.8)	32 (72.7)	0.690
History of diabetes mellitus, n (%)	408 (31.3)	101 (47.2)	52 (39.4)	17 (38.6)	0.001
History of cardiovascular disease, n (%)	271 (20.8)	56 (26.2)	34 (25.8)	13 (29.5)	0.024

*ARB* angiotensin receptor blocker, *BDI-II* Beck depression inventory-II, *BMI* body mass index, *eGFR* estimated glomerular filtration rate

Continuous variables are presented as median (IQR) or mean ± SD, appropriately. Categorical are presented n (%)

Due to missing data, the number of available information on educational background, annual income, smoking status, alcohol consumption, and history of hypertension were 1,676, 1,341, 1,676, 1,645, and 1,587, respectively

### ESKD incidence

**Fig. 2:**
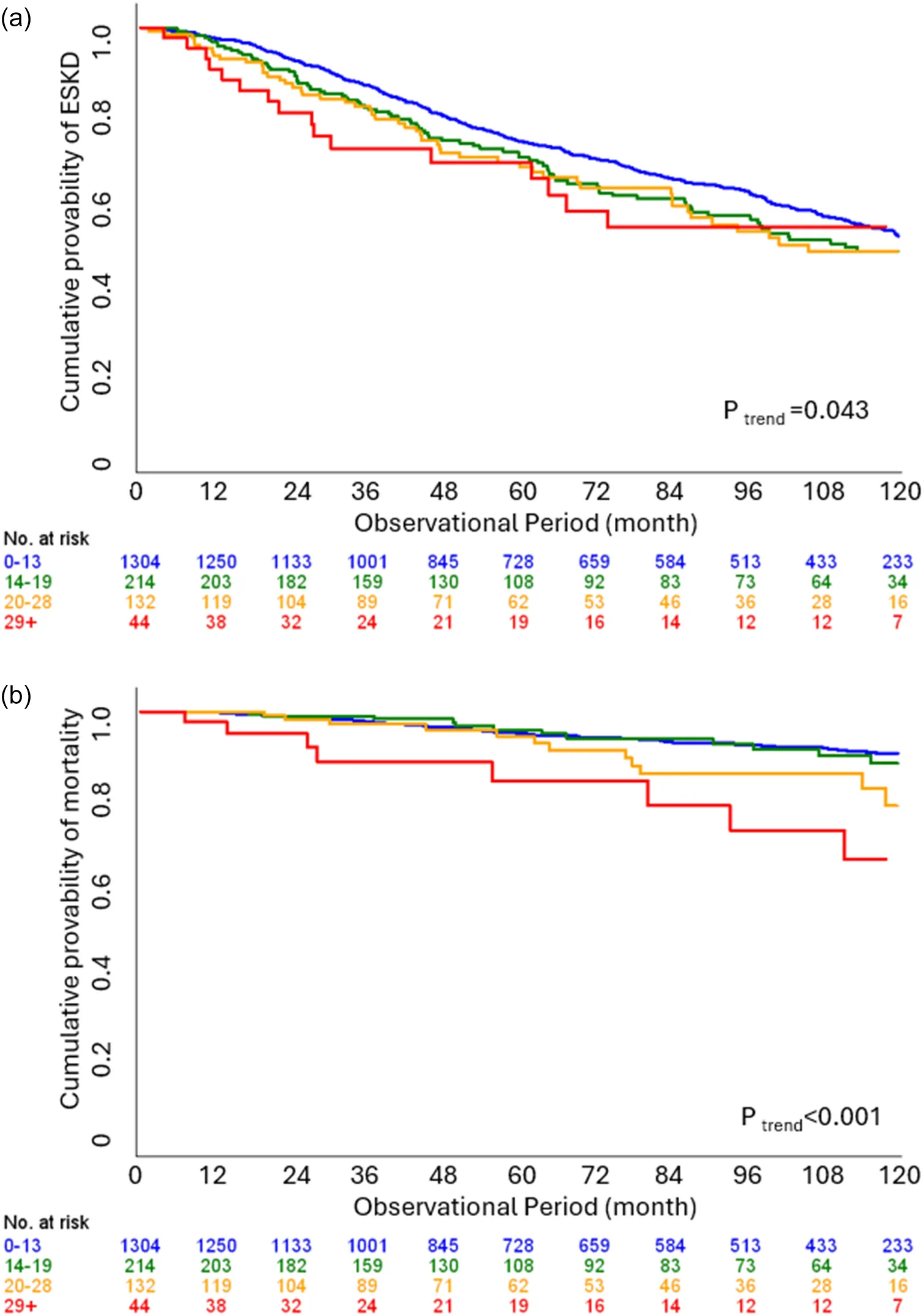
Baseline BDI-II categories and the incidence of ESKD and mortality

**Fig. 3:**
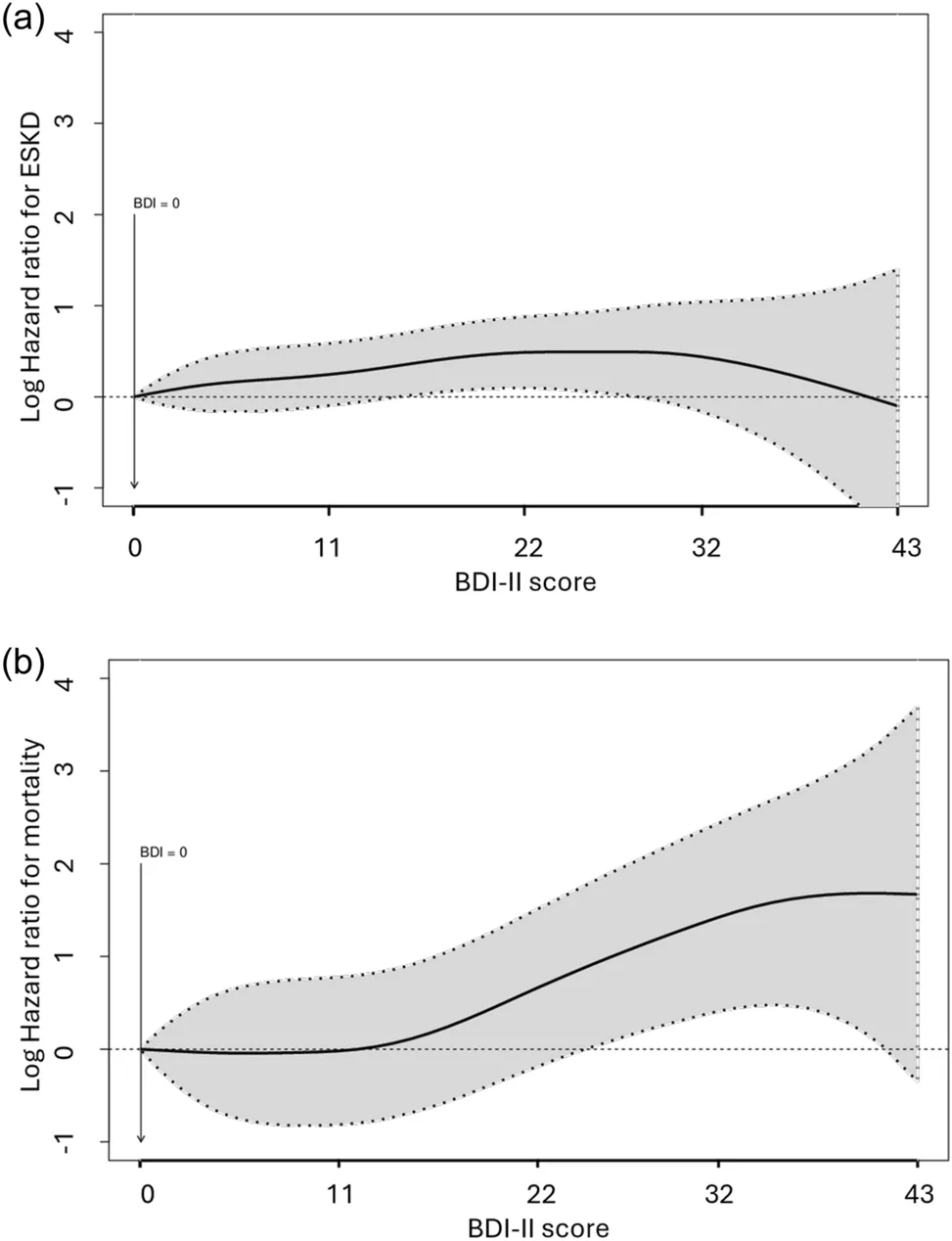
Association between BDI-II scores and the incidence of ESKD and mortality. The association between BDI-II scores and the incidence of **a** end-stage kidney disease (ESKD) and **b** mortality was evaluated using Cox proportional hazards models with penalized splines. The spline curve (solid line) represents the estimated hazard ratio across the range of BDI-II scores, while the two dotted lines indicate the 95% confidence intervals. Compared with participants with BDI-II scores of 0, those with scores of 15–28 exhibited a slightly elevated risk of developing end-stage kidney disease (ESKD), whereas participants with scores of 25–41 demonstrated a significantly higher risk of mortality

During a median follow-up of 5.7 years (IQR 3.0–9.7) in the full cohort (n = 1,694), ESKD incidence was 478 (36.7%) in the normal group, 86 (40.2%) in the mild group, 50 (37.9%) in the moderate group, and 16 (36.4%) in the severe group (Table 2). A significant trend in cumulative ESKD incidence was detected across BDI-II categories (*P*_trend_ = 0.043; Fig. 2a). To evaluate associations between BDI-II categories and ESKD incidence, CPH models were applied. The facility-stratified CPH model (Model 1; Table 3a) revealed no statistically significant associations among the mild, moderate, or severe groups compared with the normal group (HR [95%CI]: 1.00 [reference], 1.17 [0.92–1.47], 1.22 [0.90–1.63], and 1.22 [0.74–2.02], respectively). Similarly, Model 3, adjusted for baseline covariates, showed no significant associations (1.00 [reference], 1.08 [0.83–1.41], 1.22 [0.87–1.73], and 1.46 [0.80–2.68]). Nonetheless, the CPH model revealed a significant trend between BDI-II categories and ESKD incidence: P _trend_ = 0.003 in Model 1, P _trend_ = 0.004 in Model 2, and P _trend_ = 0.022 in Model 3. Additionally, CPH models with penalized splines showed a gradual increase in ESKD risk with higher BDI-II scores (Fig. 3a). Participants with BDI-II scores of 15–28 had slightly higher ESKD risk compared with those with a score of 0. Non-linearity was evaluated using a likelihood ratio test (p = 0.414), providing no evidence of non-linearity.

**Table 2:** Incidence of end-stage kidney disease and mortality across BDI-II categories

	BDI-II category			
	Normal (0–13) n = 1,304	Mild (14–19) n = 214	Moderate (20–28) n = 132	Severe (≥ 29) n = 44
End-stage kidney disease, n (%)	478 (36.7)	86 (40.2)	50 (37.9)	16 (36.4)
Incidence of end-stage kidney disease, per 100 person-year	5.94	7.00	7.22	7.42
Mortality, n (%)	77 (5.9)	13 (6.1)	13 (9.8)	8 (18.2)
Incidence of mortality, per 100 person-year	0.96	1.06	1.88	3.71

Categorical variables are presented as n (%).

**Table 3:** The association of BDI-II categories and the incidence of mortality and end-stage kidney disease

	BDI-II category				
	Normal (0–13)	Mild (14–19)	Moderate (20–28)	Severe (≥ 29)	P _trend_
	n = 1,304 HR (95%CI)	n = 214 HR (95%CI)	n = 132 HR (95%CI)	n = 44 HR (95%CI)	
a) End-stage kidney disease					
Model 1	1.00 (reference)	1.17 (0.92, 1.47)	1.22 (0.90, 1.63)	1.22 (0.74, 2.02)	0.003
Model 2	1.00 (reference)	1.08 (0.83, 1.40)	1.31 (0.94, 1.82)	1.71 (0.99, 2.95)	0.004
Model 3	1.00 (reference)	1.08 (0.83, 1.41)	1.22 (0.87, 1.73)	1.46 (0.80, 2.68)	0.022
b) Mortality					
Model 1	1.00 (reference)	1.14 (0.63, 2.05)	2.09 (1.15, 3.80)	3.54 (1.69, 7.42)	0.002
Model 2	1.00 (reference)	1.26 (0.67, 2.37)	3.20 (1.66, 6.17)	3.79 (1.62, 8.86)	< 0.001
Model 3	1.00 (reference)	1.20 (0.63, 2.27)	3.11 (1.61, 6.03)	3.51 (1.40, 8.80)	0.004

*BDI-II* Beck depression inventory-II, *HR* hazard ratio, 95%CI 95% confidence interval

Model 1 was stratified by facility. Model 2 was adjusted for age, sex, systolic blood pressure, body mass index, estimated glomerular filtration rate, log urinary albumin-to-creatinine ratio, and model 1. Model 3 was adjusted for smoking status, use of renin-angiotensin system inhibitors, and antidepressant, history of depression, diabetes mellitus, and cardiovascular disease, and model 2 (n = 1,674)

### Mortality incidence

During follow-up, mortality occurred in 77 patients (5.9%) in the normal group, 13 (6.1%) in the mild group, 13 (9.8%) in the moderate group, and 8 (18.2%) in the severe group (Table 2). A significant trend in cumulative probability of mortality was observed with increasing depression severity across BDI-II categories (P _trend_ < 0.001; Fig. 2b). To evaluate the relationship between BDI-II categories and mortality, CPH models stratified by facility were applied. In the facility-stratified CPH model (Model 1; Table 3b), higher BDI-II categories were significantly associated with mortality incidence (HR [95%CI]: 1.00 [reference], 1.14 [0.63–2.05], 2.09 [1.15–3.80], and 3.54 [1.69–7.42], respectively). After adjusting for baseline covariates, a similar association was observed in Model 3 (Table 3b): 1.00 [reference], 1.20 [0.63–2.27], 3.11 [1.61–6.03], and 3.51 [1.40–8.80]). A significant trend was observed in the association between BDI-II categories and mortality incidence using the CPH model: P _trend_ = 0.002 in Model 1, P _trend_ < 0.001 in Model 2, and P _trend_ = 0.004 in Model 3. Furthermore, CPH models with penalized splines revealed a significant J-shaped association between BDI-II scores and mortality (Fig. 3a). Participants with BDI-II scores of 25–41 showed significantly increased mortality risk compared with those with a score of 0. Non-linearity was evaluated using a likelihood ratio test (p = 0.353).

## Discussion

Over a 5.7-year period, this retrospective cohort study demonstrated that BDI-II categories were associated with the incidence of mortality and ESKD in a dose-dependent manner. In CPH models incorporating penalized splines, mortality risk showed a J-shaped association with BDI-II scores, while ESKD risk showed a gradual increase. Participants in the moderate and severe groups (BDI-II score ≥ 20) had a significantly increased risk of mortality. However, the risk of ESKD did not reach statistical significance despite a stepwise elevation in HRs with increasing depression severity. Consequently, an optimal threshold for ESKD risk could not be determined.

Trend analysis using CPH models revealed a significant dose-dependent association between depression severity and ESKD in patients with CKD. Tsai et al. reported that 428 patients with CKD and BDI-II scores ≥ 11 had significantly higher ESKD rates compared with those scoring < 11 (HR 1.50 [95%CI 1.01, 2.25]) [22]. Similarly, a prospective cohort of 262 Taiwanese patients with CKD found that depressive symptoms, diagnosed via structured interviews using the Taiwan Depression Questionnaire, independently predicted the risk of initiating KRT (HR 2.25 [95%CI 1.27, 4.98])[33]. In contrast, the CRIC study of 4,839 patients with CKD [25] and the African American Study of Kidney Disease and Hypertension (AASK) of 628 patients with CKD [34] found no significant relationship between depressive symptoms and ESKD. In the CRIC study, depression was analyzed dichotomously using a BDI-II threshold of ≥ 11, whereas the AASK study applied two cutoffs: ≥ 14 and ≥ 11. Neither study, however, examined dose-dependent associations between depression severity and ESKD incidence. Thus, the BDI-II score at which depression becomes a significant risk factor for ESKD remains uncertain. To address this, the present study categorized participants into four groups by BDI-II score and evaluated dose-dependent associations. Trend analysis using CPH models showed that ESKD incidence increased with depression severity. Likewise, spline-based CPH models revealed a dose-dependent association between BDI-II scores and ESKD incidence. The consistency between categorical and non-linear analyses underscores the robustness of the relationship between depression severity and ESKD risk.

Depression was also associated with mortality in patients with CKD in both CPH models and spline-based models. In a 3-year study of 262 Taiwanese patients with CKD, depression significantly predicted mortality (HR 3.08 [95%CI 1.69, 7.06]) [33]. Other reports, however, found no such association [22]. A meta-analysis of CKD stages 1–5 that included four small studies (n = 1,360) did not demonstrate a significant relationship between depression and mortality [35]. These discrepancies may reflect limited sample sizes and variation in assessment tools, such as the BDI-II and the Mini International Neuropsychiatric Interview (MINI). By contrast, in 3,739 CRIC participants, baseline BDI-II scores > 14 were significantly associated with all-cause mortality [21]. Subgroup analyses in that study suggested differences across racial and ethnic groups. Among White and Hispanic patients, BDI-II scores > 14 were associated with mortality, with significant results in Whites (HR 1.66 [95%CI 1.21, 2.28]) and marginal significance in Hispanics (HR 1.47 [95%CI 0.95, 2.28]). No significant association was observed among Black patients (HR 1.03 [95%CI 0.82, 1.37]). Similarly, in the AASK study, which included only Black participants, BDI-II scores ≥ 11 were not associated with mortality [34]. One possible explanation is that BDI-II scores may be influenced by cultural and racial differences in expressing and reporting depressive symptoms [36]. Addressing this issue, the present study demonstrated increased mortality risk associated with depression severity in Japanese and Asian patients with CKD living in Japan [27], using both categorical and spline-based CPH models, thereby providing more robust evidence.

This study has several limitations. First, only baseline BDI-II scores were available; follow-up assessments were not feasible. Second, 1,272 participants (42.9%) were excluded because BDI-II data were missing, as these assessments were not mandatory in the CKD-JAC study. This exclusion may have introduced selection bias. Compared with participants without BDI-II data, included participants were more often male, younger, had higher income and education, and showed lower history of DM and CVD (Supplement Table). However, history of depression and antidepressant use did not differ between groups. Although sex and younger age did not significantly modify the association between depression and mortality, individuals with higher socioeconomic status and well-controlled CVD showed decreased mortality associated with depression [35, 37]. These findings suggest that included participants may have had relatively milder disease, implying that the true effect of depression on ESKD and mortality in patients with CKD may be underestimated. Third, statistically significant trends were observed in both the log-rank test and the CPH model. However, when BDI-II categories were treated as categorical variables, no statistically significant differences in the incidence of ESKD were detected, despite consistently increasing HR with greater depression severity. This may reflect limited statistical power due to smaller sample sizes in the mild, moderate, and severe depression groups compared with the normal group. Fourth, educational background and annual income may influence depression severity and outcomes; however, these variables were expected to have substantial missing data, which could reduce the effective sample size and result in unstable HR estimates, and thus they were not included in the prespecified analysis protocol. Indeed, annual income had approximately 20% missingness overall and about 30% missingness in the severe group (13 of 44 participants), leading us to exclude it from the CPH model. Additionally, information on religion, residential area, family composition, and marital status was unavailable, although these factors may influence depression severity and outcomes [38–41]. Furthermore, while frail, malnutrition and chronic obstructive pulmonary disease may influence depression and mortality rates, it could not be included in the analysis because it was not a survey-acquired variable. Further research incorporating these variables is warranted.

In conclusion, this retrospective cohort study demonstrated that depression was significantly associated with ESKD and all-cause mortality in 1,694 patients with CKD. Future large-scale studies are required to validate the impact of depression on clinical outcomes in patients with CKD.

## Supplementary Information

Below is the link to the electronic supplementary material.

Supplementary file1 (XLSX 11 KB)

## Data Availability

Due to privacy concerns, the data supporting this article are not publicly shared but may be made available upon reasonable request to the corresponding author.
